# Second Edition of Special Issue “Strategies and Evidence in Health Communication: Evidence and Perspectives”

**DOI:** 10.3390/ijerph19031460

**Published:** 2022-01-27

**Authors:** Paolo Castiglia, Marco Dettori

**Affiliations:** 1 Department of Medical, Surgical and Experimental Sciences, University of Sassari, 07100 Sassari, Italy; castigli@uniss.it; 2 University Hospital of Sassari, 07100 Sassari, Italy

## Abstract

The second edition of this Special Issue “Strategies and Evidence in Health Communication”, published in the International Journal of Environmental Research and Public Health aims primarily to increase international literature evidence and observations in the field regarding: (i) health communication strategies and crisis communication, (ii) health education and health advocacy, and (iii) the fight against the phenomenon of Vaccine Hesitancy (VH) through training and communication activities targeting the general public and health professionals. This Special Issue builds on the premise that, despite the fact that theoretical and experimental research has contributed to an increase in knowledge and evidence about the importance of communication in healthcare, communication professionals in this field still face great challenges when trying to develop messages that effectively change the behavior of large groups of people. The need to relay fast and reliable information to the general public has therefore led public institutions to seek out new and innovative ways of transmitting health-related content. In particular, for some time now, Public Health has also been making use of the Internet and Information and Communication Technologies (ICT) to reach various population groups and achieve better health conditions for all. This practice, known as Digital Health or E-health, provides healthcare using digital tools (e.g., websites and social media networks) and easy-to-understand language. This is particularly important in the current pandemic context, where Public Health continues to face many problems and difficulties in persuading people to adhere to the guidelines issued for the containment of COVID-19, with particular reference to vaccination programs, hence the importance of acquiring and strengthening communication skills in healthcare, where correct and effective communication is immediately beneficial both to professionals and patients.

## 1. Background

Health communication, as used in the context of prevention with the aim of raising public awareness by sharing evidence-based health information, is an extremely complex issue. Public demand does not always correspond to the offer put forward by healthcare systems, and the latter are often unable to provide operational and concrete answers to users’ needs [1,2].

Attempting to change this state of affairs, counteracting misperceptions and striving to bring the HCW–patient relationship to a level of rationality and appropriateness of information is an arduous task, especially given the many determinants that play a significant role in communication processes, including (i) the completeness of the information, (ii) the conciseness of the message, (iii) the concreteness of the information supported by data and evidence, (iv) courtesy and consideration in respecting the viewpoints of others, (v) the clarity in exposition, and (vi) the correctness in the lexicon and terminology used.

Over time, there has been a shift from institutional information aimed indiscriminately at the general public to a model in which health communication sources and genres are present heterogeneously and to which various population groups have access based on their different levels of interest and education in an increasingly personal and conscious management of their own health [3,4].

In this context, a major role is played by the digital revolution which, via the new Information and Communication Technologies (ICTs), has profoundly changed the interaction between people. Today, about half of the world’s population regularly uses social media: 5.19 billion people own a mobile phone and 4.54 billion people are regularly connected to the Internet. In addition, the current COVID-19 pandemic and the consequent social isolation imposed on the population has further contributed to the increase in the use of digital platforms [5].

Moreover, the radical changes dictated by this pandemic (e.g., the spread of remote working, distance learning, and lockdown) have led the population to spend much more time on the Internet than in the past.

Furthermore, unlike in the past, nowadays, many topics are discussed at length by the general public, such as those related to health safety, vaccination prevention, antibiotic resistance, infectious diseases, non-communicable diseases, mental health, drug abuse, tobacco control, health financing, and aspects concerning the health personnel who are called to be the example through which health recommendations are made.

With the move to Web 2.0 and the social Web, the Internet user has gone from being a passive receptor with few opportunities for interaction to a more active player, providing online content of various types and in various formats (e.g., articles, comments, videos) [6]. This, together with the potential for information dissemination that the Internet provides has led to the spread of misinformation on many health-related topics.

In fact, while on the one hand, the potential offered by the Internet in the process of searching for health information has given individuals increased access to information online, on the other hand, it has exposed the user to the enormous quantity of online content and to the countless pieces of information of dubious veracity. In particular, on the subject of vaccinations, the content available online seems to increase negative attitudes (VH) more than institutional information channels that promote vaccination and offer scientifically proven information instill confidence [7,8]. This is even more worrying if one considers that the content of vaccine-related information on the web is not always well regulated and the spread of incorrect and misleading information cannot be limited or monitored.

In this regard, the dissemination of inaccurate data and misinformation on many health issues has caused damage both in terms of credibility for Public Health and in terms of trust in the proven safety, efficacy, and validity of the recommended measures of prevention [9,10,11].

Therefore, given the multiplicity and heterogeneity of the health issues facing modern society, the health workers’ technical-scientific skills alone are not sufficient to guarantee the quality of care; in fact, assisting the person requires, in addition to the care practices themselves, special attention above all to the interpersonal communication aspects. The HCW–patient relationship, which, on the one hand, sees the scientific and cognitive subordination of the patient to the healthcare provider, and on the other the complementarity of an independence whereby the patient becomes the undisputed protagonist of his or her own health and the choices relating to it, in fact, is becoming increasingly important today. In the field of vaccination communication, for example, a great deal of evidence shows that healthcare professionals play a decisive role in a consciously chosen adherence by both their patients and their colleagues [12,13].

In such a scenario, a possible intervention strategy for health promotion or disease prevention in the field of health communication could take advantage of the multiplier effect of the use of information spread on the Internet and thus more effectively meet the population’s health information needs [14]. On the other hand, the same digital communication tools in Public Health, if misused, can act as a channel for conflicting and misleading information, with a negative impact on the health choices of each individual [15,16].

In particular, during the COVID-19 pandemic, repeated media exposure to news (Infodemic), discrepancies in the number of infections and lethality figures reported in different countries, and conflicting opinions in the media about the urgent need for a vaccine made it difficult for people to find reliable sources of information [17,18,19].

From this perspective, bearing in mind the great potential offered by the Internet for those seeking health information, the use of online channels by health institutions appears necessary to ensure the dissemination of medical-scientific knowledge among users-patients in order to support citizens in making active and informed decisions regarding their own health [20,21].

In light of the above, it is essential to acquire and strengthen communication skills in the health field, where correct and effective communication, alongside the building of effective professional relationships (with the person to whom the health intervention is addressed at its center), is an immediate health benefit.

## 2. Conclusions

One of the major challenges Public Health will face in the coming decades is the fight against misinformation. Strong multidisciplinary alliances among health professionals, the development of health communication and education activities using all available information methods (including innovative ICTs), and occasions for a two-way dialogue between HCWs and patients are just some of the possible strategies that should be considered by international and national health authorities and agencies in order to reinforce health messages to promote self-care and the adoption of correct health behaviors.

To this end, the Special Issue “Strategies and Evidence in Health Communication”, in its first edition, with 14 manuscripts published online by 31 March 2021, put forward many interesting contributions and insights to the scientific community. However, the current health emergency has further highlighted that, although patient empowerment and users’ abilities to self-manage their search for health information can be useful in terms of health literacy and proper management of their own health, the presence of the health professional as a health communicator is essential to guide users towards accurate and scientifically proven information.

The second edition of the Special Issue will, therefore, provide a useful tool for the scientific community to discuss these topics in greater depth also within the context of the current pandemic.
